# Diagnostic and Therapeutic Management of the Thoracic Outlet Syndrome. Review of the Literature and Report of an Italian Experience

**DOI:** 10.3389/fcvm.2022.802183

**Published:** 2022-03-22

**Authors:** Giuseppe Camporese, Enrico Bernardi, Andrea Venturin, Alice Pellizzaro, Alessandra Schiavon, Francesca Caneva, Alessandro Strullato, Daniele Toninato, Beatrice Forcato, Andrea Zuin, Francesco Squizzato, Michele Piazza, Roberto Stramare, Chiara Tonello, Pierpaolo Di Micco, Stefano Masiero, Federico Rea, Franco Grego, Paolo Simioni

**Affiliations:** ^1^Angiology Unit, Department of Cardiac, Thoracic and Vascular Sciences and Public Health, Padua University Hospital, Padua, Italy; ^2^Department of Emergency and Accident Medicine, Hospital of Treviso, Treviso, Italy; ^3^Physical Medicine and Rehabilitation Unit, Department of Neurosciences, Padua University Hospital, Padua, Italy; ^4^Thoracic Surgery, Department of Cardiac, Thoracic and Vascular Sciences and Public Health, Padua University Hospital, Padua, Italy; ^5^Vascular Surgery, Department of Cardiac, Thoracic and Vascular Sciences and Public Health, Padua University Hospital, Padua, Italy; ^6^Department of Medicine DIMED, Institute of Radiology, Padua University Hospital, Padua, Italy; ^7^Unit of Advanced Clinical and Translational Imaging, Department of Medicine, University Hospital of Padua, Padua, Italy; ^8^Department of Internal Medicine and Emergency Room, Naples Buon Consiglio Fatebenefratelli Hospital, Naples, Italy; ^9^Department of Internal Medicine, General Medicine Unit, Thrombotic and Haemorrhagic Disorders Unit, University Hospital of Padua, Padua, Italy

**Keywords:** diagnosis, treatment, thoracic outlet syndrome, surgery, rehabilitation

## Abstract

The Thoracic Outlet Syndrome is a clinical potentially disabling condition characterized by a group of upper extremity signs and symptoms due to the compression of the neurovascular bundle passing through the thoracic outlet region. Because of the non-specific nature of signs and symptoms, to the lack of a consensus for the objective diagnosis, and to the wide range of etiologies, the actual figure is still a matter of debate among experts. We aimed to summarize the current evidence about the pathophysiology, the diagnosis and the treatment of the thoracic outlet syndrome, and to report a retrospective analysis on 324 patients followed for 5 years at the Padua University Hospital and at the Naples Fatebenefratelli Hospital in Italy, to verify the effectiveness of a specific rehabilitation program for the syndrome and to evaluate if physical therapy could relieve symptoms in these patients.

## Introduction

The Thoracic Outlet Syndrome (TOS) is a clinical potentially disabling condition characterized by a group of upper extremity signs and symptoms due to the compression of the neurovascular bundle passing through the thoracic outlet region, an anatomical site enclosed among the anterior middle scalene muscles, the clavicle, and the first rib.

According to the pathophysiology, TOS can be classified in: neurogenic (nTOS), arterial (aTOS), and venous (vTOS). Each one of these may recognize either a congenital (cervical ribs or anomalous first rib), or traumatic (whiplash injuries, falls), or functionally acquired (active and vigorous repetitive sport- and/or work-related activities) cause (1–4).

The incidence of TOS ranges from 3 to 80/1,000 people; nonetheless, due to the non-specific nature of signs and symptoms, to the lack of a consensus for the objective diagnosis, and to the wide range of etiologies, the actual figure is still a matter of debate (1–4).

In 2016, the Society for Vascular Surgery issued a consensus document attempting to standardize the terminology, definitions, diagnostic criteria, reporting standards and therapeutic options for each type of TOS (1).

This article summarizes the current evidence about the epidemiology and the pathophysiology of TOS, and its diagnostic and therapeutic approach at the Padua University Hospital and at the Naples Fatebenefratelli Hospital in Italy together with a report of our personal experience in this setting.

### Epidemiology

nTOS accounts for 90–95% of cases, vTOS for 3–5%, and aTOS for the remaining 1–2%. Signs and symptoms most often occur between 20 and 50 years and are usually unilateral, especially involving the dominant arm. While nTOS is more prevalent in women, aTOS equally affects both genders, and vTOS is more frequent in men. Both aTOS and nTOS share common etiologies causing artery and/or nerve compression, such as trauma (whiplash injury), or anatomic abnormalities (cervical ribs, anterior and/or middle scalene hypertrophy, tumors, or fibrous bands). vTOS is more common in athletes (e.g., volley, baseball, swimming, body-building, etc.), manual workers or subjects performing vigorous activity (2–6).

### Anatomy

The superior thoracic outlet is the anatomical area crossed by the brachial plexus, and by the subclavian artery and vein. It lies between the anterior and middle scalene muscles, superiorly to the first rib, posteriorly to the clavicle, laterally to the sternal manubrium.

The brachial plexus is formed by the anterior branches of cervical roots C5 to C8, the anterior branch of the first thoracic nerve (T1) and the anastomotic branches of C4 and T2. It supplies nerve fibers to the thorax and upper limb.

**Figure 1:**
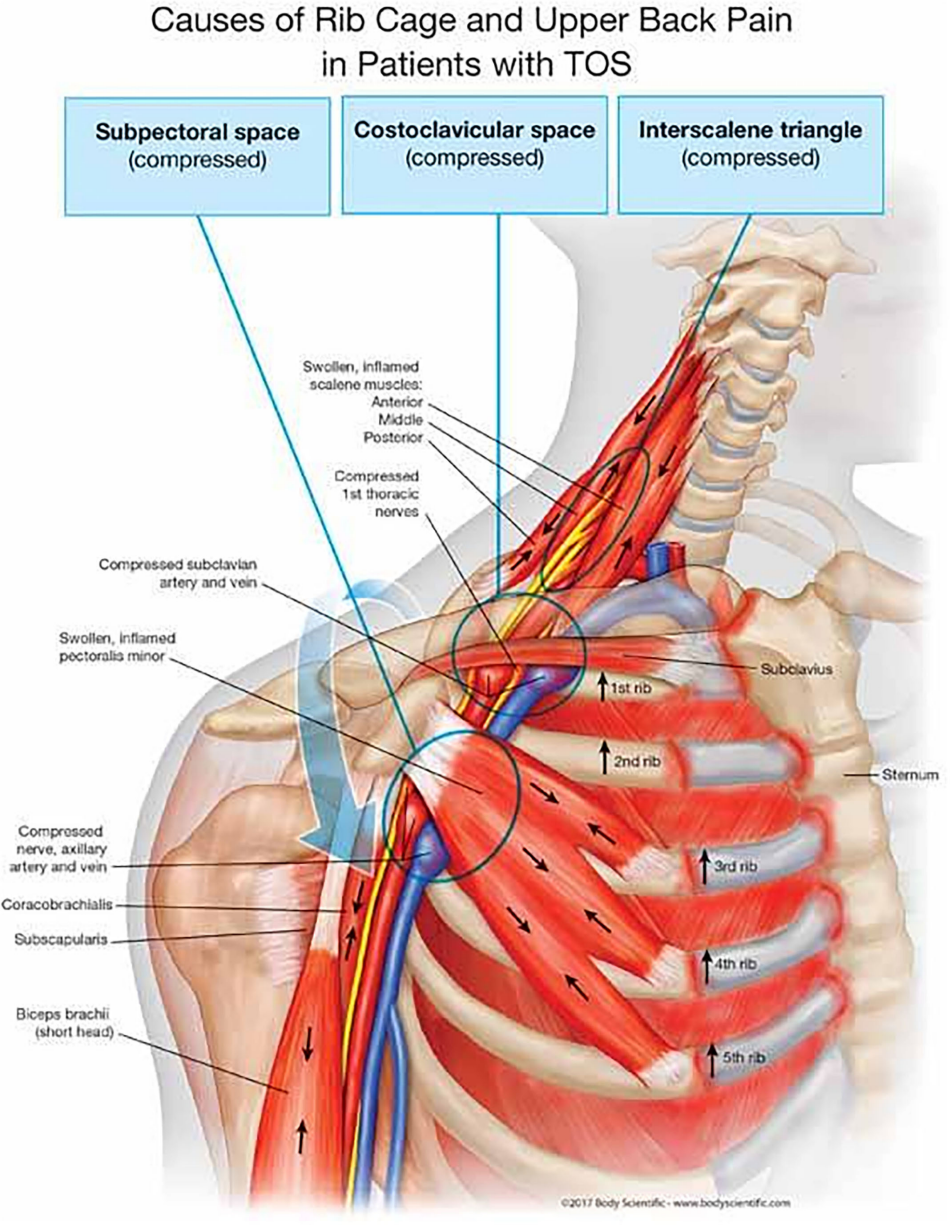
Anatomical spaces involved in TOS. Originally published in “The human spring approach to thoracic outlet syndrome”. Author: Dr. James Stoxen DC FSSEMM (hon); illustrated and copyrighted by Body Scientific International, LLC – www.BodyScientific.com. Permission of the Author with agreement signed in February 2022.

The thoracic outlet includes three distinct anatomical spaces where a compression of the neurovascular structures may occur (Figure 1):

- *Triangle of the scalenes*: most frequently involved in the compression of the brachial plexus and of the subclavian artery. It is formed anteriorly by the anterior scalene muscle, and posteriorly by the middle scalene muscle; the base of the triangle is made up by the first rib.
- C*osto-clavicular space*: most frequently involved in the compression of the subclavian vein. It is outlined anteriorly by the middle third of the clavicle, and postero-medially by the first rib and by the aponeurosis of the subclavian muscle.
- S*ubpectoral space*: the entire brachial plexus may be compressed at this level (5, 7). It is bordered anteriorly by the tendon of the pectoralis minor muscle and the coracoid process, and posteriorly by the thoracic wall.

### Pathophysiology

A list of the most common congenital or acquired causes of TOS is reported in Table 1 (8–12).

**Table 1:** Common causes of TOS.

**Congenital factors**	**Acquired abnormalities**
Cervical rib	Postural factors
1st rib anomaly	Fall injuries to upper limb
C7 transverse process abnormalities	Clavicular Fracture
Fibrous bundles between transverse process of C7 and the 1st rib	1st rib fracture
Supernumerary rib	Whiplash injury
Anomalies of scalene muscle insertion	Repetitive stress injuries
Supernumerary scalene muscle	Hypertrophy of the scalene muscles
Exostosis of the first rib	Decrease trapezius, scapulae elevator, rhomboides muscles tone
Cervicodorsal scoliosis	Shortening of the scalene, trapezius, elevator scapulae, pectoralis muscles

#### Congenital Factors

The prevalence of a cervical rib accounts for 1–2% in the general population, women being most frequently affected. About 20% of nTOS cases are attributable to this anomaly, that also constitutes a risk factor for the development of aTOS (9, 10).

#### Acquired Factors

Acquired causes of TOS include: consolidation defects of the first rib and of the clavicle, and muscular hypertrophy due to physical or professional repetitive activities involving lifting weights. Also, postural disorders and scapular girdle dysfunction may lead to narrowing of the costoclavicular angle, resulting in compression of the neck vascular bundle (13). Acute symptoms may develop following traumatic events, such as whiplash or a fall on an outstretched arm (14). Other causes are Pancoast tumor, hereditary multiple exostosis, and osteochondromas.

## Clinical Diagnosis

### Clinical Features and Physical Examination

It is essential to collect an accurate medical history describing symptoms, their onset and duration, the presence of aggravating/alleviating factors, and the associated degree of disability. Pain in the neck, occipital region, chest, shoulders and upper limbs is frequently reported. Other symptoms are: paraesthesia, hypoesthesia or anesthesia, weakness, heaviness, dyschromia and dystermia (5, 15, 16). A complete list of TOS-related signs and symptoms is reported in Supplementary Table 1S (see Supplementary Material).

### Provocative Maneuvers

These maneuvers (see Supplementary Table 2S) may evoke TOS typical symptoms (10). Simultaneous positivity of several maneuvers may increase specificity; for instance, in a study by Gillard et al. the specificity of the Adson and Roos tests ranged from 30 to 72% when used alone, increasing to 82% when both were performed (5, 17–20).

## Imaging

### Plethysmography

Finger plethysmography may detect a subclavian artery compression displaying both a delayed upslope of the sphygmic wave, and a loss of the dicrotic notch during provocative maneuvers. These findings, however, are also common in the normal population, mandating further diagnostic testing (1, 4, 21).

### Ultrasound Assessment

In our Institutions, patients with suspected vascular TOS undergo dynamic bilateral Color-Coded Doppler Ultrasonography (CCDU). CCDU allows for non-invasive real-time visualization, as well as for pulsed Doppler waveform analysis and blood-flow velocity evaluation, of the subclavian and axillary vessels, both at rest and during provocative maneuvers (1, 21–23). Currently, a consensus on the preferred technique to evaluate suspected TOS by ultrasonography is lacking. In our Institutions, all sonographic examinations are performed with last generation ultrasound equipment, using a 3–13 MHz multifrequency linear-array probe for higher accuracy, according to a standardized technique (see Section 1, Supplementary Material).

### Chest and/or Cervical X-Ray

Plain chest and/or cervical X-ray films should be obtained in all patients with suspected TOS, being a sensitive and low-cost modality to identify major bone abnormalities potentially causing TOS (Table 1). In doubtful cases, contrast-enhanced computed tomography angiography (CTA) or magnetic resonance angiography (MRA) may be performed, as they provide a more detailed survey of the anatomy, especially useful to diagnose TOS subtypes (21, 24–26).

### CTA

Detailed methodology is reported in Section 2 of the Supplementary Material.

CTA is especially useful in symptomatic patients without skeletal anomalies at conventional radiography (27). Several studies have focused on the efficacy of 3-D reconstructions, which can define the anatomical details, highlight anatomical relationships between vascular and bone structures, and allow surgery planning.

CTA can detect deep-vein thrombosis and venous collateral circuits, both consequences of venous compression. CTA is the preferred choice for the evaluation of patients with suspected anomalous ribs or fractures, and it is also useful in postoperative patients with suspected remnant 1st rib (Supplementary Figure 1S).

Among CTA limitations are: ionizing radiation exposure, scarce visualization of brachial plexus, mandatory supine position, and poor arm/shoulder hyperabduction due to the gantry size (28, 29).

### MRA

Detailed methodology is reported in Section 3 of the Supplementary Material.

MRA with provocative maneuvers is the cross-sectional imaging test of choice in patients with suspected TOS, allowing for careful study of all the anatomical components of the thoracic outlet (30). Even without contrast media, MRA can show arterial and neural plexus compression, venous thrombosis and venous collateral circuits, muscle hyperthrophy and hypotrophy, accessories muscles, and anomalous fibrous bands (Supplementary Figure 2S). T1-weighted sagittal sequences are useful for assessing vascular and neural compression and for revealing post-stenotic dilation; instead, coronal sequences supply good views of the brachial plexus, and may highlight fibrous bands. All sequences should be acquired during bilateral arm abduction with the head and neck in the neutral position, and repeated during arm adduction with additional contrast administration (31–34).

MRA has some advantages over CTA, such as multi-planar analysis, optimal small parts visualization, and lack of ionizing radiation, the latter being of particular interest in the generally young patient population affected by TOS. However, when MRA cannot be performed due to claustrophobia or implanted devices, CTA remains the study of choice.

Both MRA and CTA are difficult to perform in patients with severe or dialysis-dependent renal failure.

### Venography and Arteriography

Both venography and arteriography have been historically considered the diagnostic “gold standard”, but due to single-plane resolution and invasiveness, they have been widely replaced by CTA and MRA, and currently have a very limited use (e.g., severe arterial insufficiency or ischemia, aneurysms, extended thrombosis or vein fibrosis; see Supplementary Figure 3S) (2, 21).

## Electrodiagnostics

True nTOS is a plexopathy caused by a fibrous band from a rudimentary cervical rib to the first thoracic rib, trapping the lower trunk of the brachial plexus and developing a sensory and motor deficit in the C8-T1 distribution territory. nTOS is often confused clinically with ulnar neuropathy at the elbow, or with C8-T1 radiculopathy (35). In nTOS, T1 fibers tend to be preferentially affected, leading to a distinctive pattern. In most cases, the ulnar sensory nerve action potential (SNAP) amplitude is low but not absent, the medial antebrachial cutaneous SNAP amplitude is also usually low or absent, and the median SNAP is normal. Additionally, the axonal loss is characterized by a low compound muscle action potential (CMAP) amplitude in both the median and ulnar motor nerves, typically with a more profound decrease in the median response affecting the median-innervated thenar muscles (5, 36). Needle electromyography abnormalities are found in median- more than in ulnar-innervated C8–T1 muscles, and less so in radial innervated C8 muscles; in particular, the thenar muscles are more severely involved than the hypothenar ones. Such abnormalities may include fibrillation potentials, positive sharp waves, and long duration motor unit potentials (MUPs). The abductor pollicis brevis is preferentially innervated by the T1 root, and is most commonly affected in nTOS (37).

## Treatment

Initial management of TOS is usually conservative (dedicated physical therapy, addressing muscle imbalance, postural abnormalities and neural mobilities). Patients are taught that overhead activity, heavy lifting, repetitive motions or use of vibratory tools will aggravate their symptoms, and play against good long-term physical or surgical results.

Although a consensus on the appropriate conservative regimen for nTOS remains controversial, a multimodal approach including patient education, TOS-specific rehabilitation and drug therapies has shown positive results in 60–70% of cases.

If symptoms persist after at least 3–6 months of rehabilitation and patients are experiencing some degree of disability at work, sleep, recreation, or daily living activities, a surgical approach should be considered, and treatment choice is usually related to surgeon experience, kind of involved anatomical district, extent of surgical procedure, and exposure needs (38, 39). Other indications for surgery include arterial and/or venous compression with or without parietal damages, thrombosis or aneurysms.

### Medical Therapy

Pharmacological interventions often provide symptom relief, and mainly include analgesics (non-steroidal anti-inflammatory drugs and/or opioids) for neuropathic pain, as well as muscle relaxants, anticonvulsants and/or antidepressants as adjuvants.

#### Parenteral Treatment

Symptomatic patients with scalene muscles contracture who fail to respond to conservative approach may benefit from botulinum toxin injection (BTX-A), though its efficacy is still controversial. Some studies suggest that BTX-A injections are associated with significant pain and symptoms reduction in up to 70% of patients, for up to 3 months. Injection of steroids and local anesthetics (bupivacaine, lidocaine, triamcinolone and ropivacaine) has also shown good clinical efficacy (40, 41).

#### Conservative Rehabilitative Treatment

The main purpose of rehabilitative treatment is to restore the width of anatomical spaces, whose compression is at the basis of the pathology; moreover, rehabilitation treatment can support the diagnosis of TOS, if symptom improvement is observed. Physical therapy is associated with significant symptom improvement in 50 to 90% of patients (42).

There is lack of consensus on the duration and the timing of the rehabilitation process, even within the TOS subtypes, according to the degree of disability and other individual factors. Revaluation and adaption of therapy must be ongoing, and dictated by symptoms status. A 6-month physical therapy program consisting of home-exercises, stretching, postural corrections, and muscle recruitment patterns, primarily focusing on the neck and shoulder, can alleviate symptoms associated with TOS. Generally, patients with mild TOS are expected to improve within 6 weeks (43, 44). In refractory cases undergoing decompressive surgery, post-surgical rehabilitation plays a key role in the recovery of autonomy and upper limb range of motion, and in the improvement of the patients' quality of life (45–47).

Key points of rehabilitative treatment are: postural education (e.g., avoid carrying heavy weights and prolonged hyperabduction of the upper limbs); cervico-dorsal and scapular girdle massage (to resolve contractures); diathermy or laser therapy (for antalgic purposes); kinesiotherapy (to restore the balance between muscles opening and closing thoracic egress).

The rehabilitation course should be scheduled as follows: postural exercises; static reinforcement of the muscles that open the strait; stretching of the muscles that close the strait; kinesiotherapy of the cervical spine; breathing exercises to lessen the overload of scalene muscles and to lower the first rib.

The rehabilitation program must be guided by a physiotherapist specialized in TOS treatment (see Section 4 in Supplementary Material for details) (43, 44). A summary of exercises targeting the shoulder muscles are shown in Supplementary Figure 4S and Supplementary Table 3S.

## Surgical Treatment

### Thoracic Surgery

With proper and accurate patient selection and compliance, the surgical management of TOS may have excellent outcomes. The various syndromes are similar and the specific compression mechanism is often difficult to identify; however, the first rib seems to be a common denominator along which most compressive factors operate (Supplementary Figure 5S) (41, 48, 49).

Many authors think that resection of the first rib, with cervical rib when present, is best performed through the trans-axillary approach (see Section 5, Supplementary Material) for complete removal with subclavian vascular decompression, while the supraclavicular approach (see Section 6, Supplementary Material) is often preferred in nTOS, but may be appropriate in any combinations of these clinical syndromes (50, 51).

In properly selected patients, clinical results of first rib resection may be considered good (complete relief of symptoms) in 85% of patients, fair (improvement with some residual or recurrent mild symptoms) in 10% and poor (no change from preoperative status) in 5% (50, 52, 53).

Recently, removal of the first rib on total videothoracoscopic or robotic approach was described, but the outcomes are yet to be completely determined (51, 54).

Considering the peculiar anatomical district, there are many possible complications and, although rarely, they may also be very serious (55, 56).

Among these are:

- *Brachial plexus injury*, due to excessive traction to the roots of the plexus during mobilization of first rib; to reduce this risk, it is useful to raise the shoulder and to bend the head toward the operative side.
- *Phrenic nerve injury*, may occur with just minor traction or during a lifting with a forceps, so every contact should be avoided or limited, even with a vessel loop. Another kind of damage is the contact with the cautery, uni- or bipolar.
- *Long thoracic nerve injury*, may occur by cutting one of branches of the nerve, usually running near the lateral side of middle scalene muscle, causing a winged scapula.
- *Thoracic duct injury*, the thoracic duct may lie in the middle of the scalene fat pad in the lower left portion of the neck; injury at this level causes milky (or clear) fluid leaking in the operative field. If a leak is evident, damage is managed by ties, clips or bipolar cautery.
- *Vascular injury*, an injury to the subclavian artery or vein may occur, that can be more easily controlled through the supraclavicular approach.

Based on this consideration, a thoughtful, well-articulated, informed consent is mandatory.

### Vascular Surgery

The three main concepts of vascular surgical treatment are: relieving the arterial compression (the trigger of the disease), repairing the damaged subclavian artery (local complication), and restoring the distal circulation (distal complication) (57).

The indications for vascular surgery are: failure of conservative therapy with persisting disabling symptoms that interfere with daily life activities; or with vascular (arterial) complications: stenosis, thrombosis, distal embolization or aneurysm.

Transaxillary first rib resection (as originally described by Roos) (2) is the gold standard for the treatment of aTOS (see Section 5, Supplementary Material) (58). The rationale of first rib resection is that it guarantees a decompression of the neurovascular bundle in all cases of costoclavicular space narrowing (59). Roos rib resection seems to be more effective in preventing symptoms recurrence compared to scalenectomy, because also in those cases of anterior scalene hypertrophy/anatomic variation, TOS is still determined by the reduction of the costoclavicular space, that is corrected by the resection of the first rib (Supplementary Figure 5S) (59). Transaxillary rib resection carries the disadvantage of a limited subclavian artery exposure, therefore other access (typically supra-clavicular) are needed if an arterial intervention is needed.

#### Surgical Treatment of the Damaged Artery

Subclavian artery impingement may occasionally result in local arterial complications, such as stenosis or chronic occlusion, post-stenotic dilatation or aneurysm formation (60).

Subclavian/axillary artery stenosis or occlusion may be the consequence of the chronic mechanical stress at the level of the costo-clavicular space. This may rarely result in chronic upper limb ischemia with claudication, rest pain or ischemic tissue loss. The gold standard for treatment in these cases is represented by surgical by-pass. The by-pass sources of inflow and outflow depend on the specific anatomical situation, that is preoperatively planned according to the CTA/MRA or arteriography.

In case of subclavian artery aneurysm, the rational for the surgical treatment is to eliminate the source of chronic embolization. Surgery consists in arterial resection and substitution with a vascular graft, performed via a supraclavicular approach. The preferred conduit in this case is represented by heparinized polytetrafluorethylene (PTFE), anastomosed in an end-to-end fashion (60, 61).

There have been scattered reports of endovascular repair of the subclavian artery combined with surgical decompression of the thoracic outlet and more data are needed to assess the role of endovascular solutions in aTOS (57).

### Restoration of the Distal Circulation

#### Acute Limb Ischemia

The clinical presentation is characterized by pulselessness, acute pain, pallor, paresthesia, paralysis or paresis, and hypothermia of the hand. This clinical situation warrants an emergent treatment to preserve the hand function and viability. Acute limb ischemia in aTOS may be related to subclavian artery thrombosis, embolization from the subclavian artery (due to presence of an aneurysm or arterial wall thrombosis), or both. The treatment is typically based on Fogarty thrombo-embolectomy and/or catheter-directed thrombolysis (61).

Fogarty thrombo-embolectomy is performed through an oblique incision at the level of the cubital fossa. The bicipital aponevrosis is divided exposing the distal brachial artery and its bifurcation into the radial and ulnar arteries. A transverse arteriotomy is performed and the Fogarty catheter is advanced through the proximal and distal arterial axis.

Fogarty thrombo-embolectomy is effective in restoring the patency of acutely occluded arteries; however this may not be always sufficient in aTOS, where microembolization may be responsible for distal circulation impairment. Distal bypasses are sometimes necessary in patients with chronically occluded arteries of the upper limb due to chronic embolization from aTOS. Intra-arterial thrombolysis is based on loco-regional infusion of thrombolytic agents (typically urokinase) through a multi-hole catheter placed at the level of the arterial thrombosis. It has the advantage to be effective also on smaller distal vessels that are not affected by surgery, but it may take 12–72 h to achieve an optimal result, therefore its use alone is not recommended in acute limb ischemia with threatened limb. In our experience, we use thrombolysis after Fogarty thrombo-embolectomy, in those cases where surgery alone is not sufficient to restore an adequate blood flow, because of distal arterial branches (i.e., interdigital) occlusion. In any case, revascularization does not eliminate the cause of arterial compression, thus physical therapy or/and surgical first rib resection are still indicated after the acute event.

#### Chronic Limb Ischemia

Chronic distal embolization from the damaged subclavian artery may determine chronic occlusion of the arteries of the arm or forearm. In case of disabling claudication, rest pain, or tissue loss, a peripheral revascularization is indicated. This typically consists in a surgical bypass; also in this case the precise inflow and outflow sources depend on the specific case. If available, a saphenous vein graft is preferred in this anatomical region (60–62).

## Personal Experience

### Study Cohort and Methods

In 2019 we started a retrospective survey to verify the effectiveness of a specific rehabilitation program for TOS, and to evaluate if physical therapy could relieve symptoms of TOS. We assessed 324 patients referred to our Institutions between 2004 and 2019, 270 females (83%) and 54 males (17%), aged between 12 and 59, with an average age of 38 years (SD 12). Data were collected from patients attending the outpatient clinic of the Clinic of Physical and Rehabilitative Medicine, Thoracic Surgery, Angiology and Occupational Medicine. Patients were classified on the basis of job categories, and of TOS subtype: aTOS 4%; vTOS 7%; vascular TOS (venous and arterial) 13%; nTOS 29%; miscellaneous TOS 47%. The following comorbidities were recorded in our cohort: C7 abnormalities (15%); shoulder disorders (i.e.: rotator-cuff tendinopathies, impingement syndrome, or other) (14%); history of whiplash (13%); previous episodes of deep venous thrombosis of the upper limbs (24%). All patients underwent diagnostic imaging procedures, such as cervical spine radiograms, basal and contrast-enhanced cervical CTA/MRA, CCDU, and electromyography.

All 324 patients were offered a specific rehabilitation protocol in appropriately trained centers, 285 (88%) of them accepted, and 39 (12%) refused any type of treatment. Patients rejecting treatment were much alike patients who underwent rehabilitation (Table 2), but declined because they either could not afford enough time to follow the complex rehabilitation program, or had geographic inaccessibility to the rehabilitation centers who were chosen for the study.

**Table 2:** Demographics of the investigated population.

		**Treatment**		
		**No (39)**	**Yes (285)**	***p***
Age	(years, mean + SD)	39.6 ± 11.7	37.9 ± 11.7	0.405
Sex	F	32 (82.1)	238 (83.5)	0.820
	M	7 (17.9)	47 (16.5)	
TOS variant	aTOS	1 (2.6)	12 (4.2)	0.254
	vTOS	1 (2.6)	22 (7.7)	
	vaTOS	4 (10.3)	38 (13.3)	
	nTOS	17 (43.6)	77 (27.0)	
	mTOS	16 (41.0)	136 (47.7)	
Job	High risk workers^a^	9 (23.1)	101 (35.4)	0.151
	Low risk workers^b^	30 (76.9)	184 (64.6)	
Comorbidities	C7 abnormalities	6 (15.3)	42 (14.7)	0.991
	Shoulder disorders	6 (15.3)	39 (13.7)	
	Whiplash	5 (12.8)	36 (12.6)	
	Previous dvt upper limbs	9 (23.1)	68 (23.9)	
Conservative treatment	Massages		20 (7.0)	
	Massages + specific TOS m&ph rehab protocol		74 (26.0)	
	CTEN stimulation		17 (6.0)	
	CTEN stimulation + specific TOS m&ph rehab protocol		60 (21.1)	
	hydrogalvanotherapy		15 (5.3)	
	Hydrogalvanotherapy + specific TOS m&ph rehab protocol		53 (18.6)	
	Specific TOS m&ph rehab protocol		46 (16.1)	
Surgical Treatment	Cervical rib resection		4 (1.4)	
	Cervical rib resection + neurolysis		3 (1.1)	
	Cervical rib resection + scalenectomy		2 (0.7)	
	First rib resection		11 (3.9)	
	First rib resection + neurolysis		2 (0.7)	
	First rib resection + scalenectomy		1 (0.4)	
	Neurolysis		1 (0.4)	
	Other surgery		3 (1.1)	
	Scalenectomy		2 (0.7)	

*^a^Video-terminalists, clerks, teachers.*

*^b^Other jobs*.

Treated and untreated patients were evaluated by the Numeric Pain Rating Scale (NRS) to assess pain burden, either at baseline (T0), after 6 months (T1), and at the last available follow-up visit (T2). Three groups of patients were identified: worsened symptoms (NRS value at T-2 greater than at T-0); stationary symptoms (no difference between T-2 and T-0 NRS values); improved symptoms (NRS value at T-2 lower than at T-0). All data were compared by Chi-square test, Fisher-Freeman-Halton exact test or Student' *t*-test, where appropriate. The effect of treatment on the temporal trend of NRS-score was evaluated by repeated measures analysis of variance (ANOVA) using NRS-scores at T0, T1, and T2 as within-subjects factor, and treatment as between-subjects factor.

## Results

**Figure 2:**
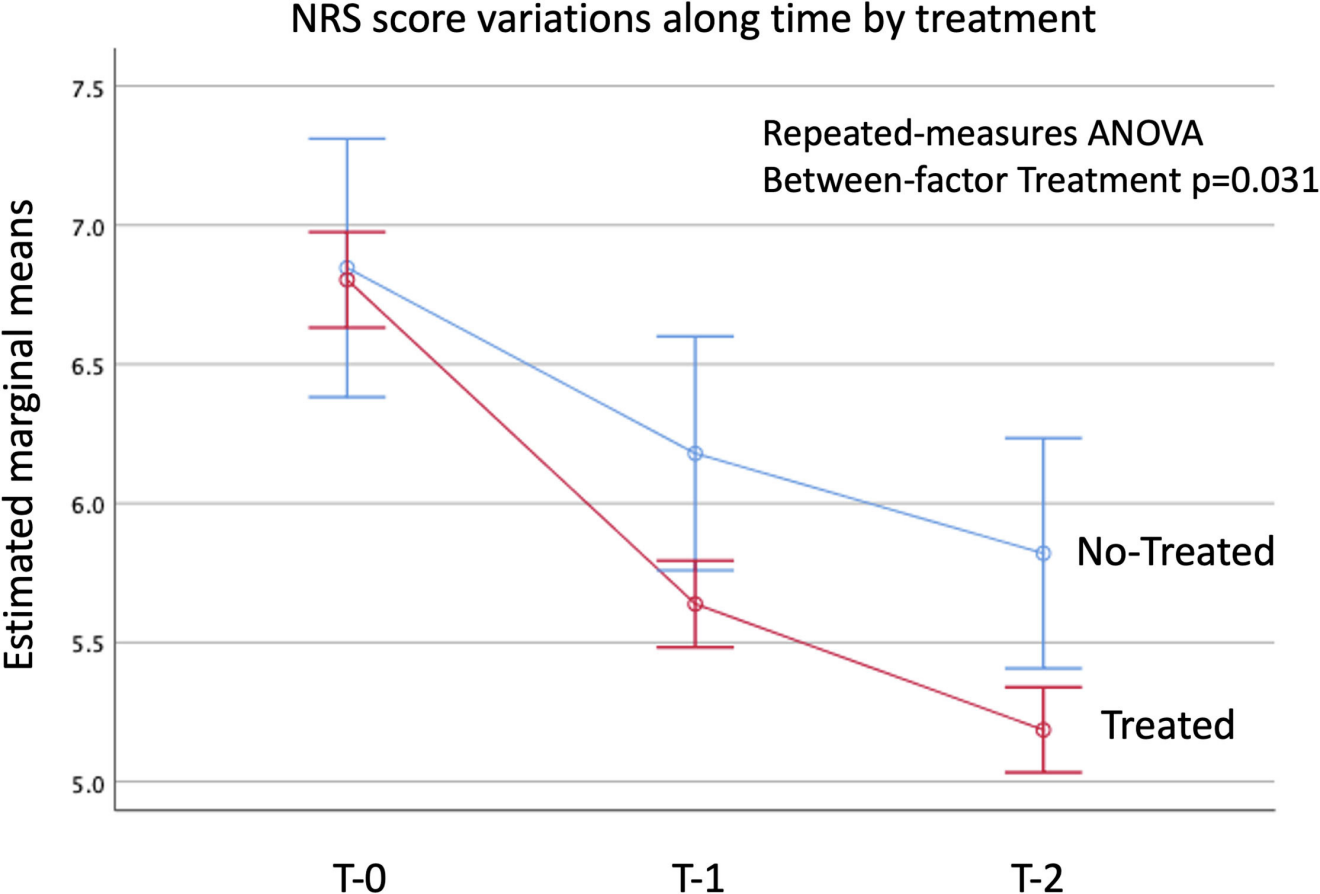
Estimated marginal means of the NRS-score at T0, T1, and T2. Vertical bars denote the 95% C.I. of means.

The patients' characteristics are summarized in Table 2. Overall, a statistically significant higher number of patients undergoing a specific rehabilitation protocol reported either improved or stationary symptoms as compared to untreated patients, at the last available follow-up visit (T2) (Figure 2). Namely, of 285 patients in the TOS specific rehabilitative program, 192 (67%) had improved symptoms; 72 (25%) were stationary; and 21 (7%) had worsened symptoms at T-2 (Table 3; *p* < 0.01). A detailed description of NRS scores at the three time-points in the two patient groups is reported in Supplementary Table 4S.

**Table 3:** Symptoms variation in patients undergoing a specific rehabilitation protocol vs. those refusing treatment at the last available follow-up visit.

	**Control group** **(39)**	**TOS specific** **rehabilitation protocol** **(285)**	*p* value*
Improved, *n* (%)	21 (53.8%)	192 (67.4%)	<0.01
Stationary, *n* (%)	0	72 (25.3%)	
Worsened, *n* (%)	18 (46.2%)	21 (7.4%)	

*Fisher-Freeman-Halton exact test.

Worsened symptoms occurred more frequently in workers of the services industry (computer users, teachers, clerks), as compared to other workers at lower risk of TOS (*p* = 0.046); and in patients with shoulder disorders as compared to those without (*p* = 0.04) (see Supplementary Table S5). On the contrary, a history of C7 abnormalities or of whiplash did not impact on symptoms. Of the 285 patients participating in the rehabilitation protocol, 19% underwent hydro-galvanotherapy, massages, or cervical transcutaneous electrical nerve stimulation only; while 81% had one or more of the aforementioned interventions plus a specific TOS manual and physical rehabilitation training.

Only 29 (10%) out of 285 patients, all 21 patients with worsened symptoms plus 8 patients with stationary symptoms underwent surgical treatment (single or combined surgical procedures). Of them, 50% underwent first rib resection, 30% cervical rib resection, 20% scalenectomy, 20% neurolysis; and 10% other surgery.

Interestingly, no significant differences in terms of NRS scores were found at T-2 between patients who underwent surgery vs. patients who did not (Supplementary Table S6).

## Comment

Our results emphasize the importance of rehabilitative treatment as a first-line therapeutic approach in the management of patients with TOS. Indeed, according to the NRS scores provided by our patients, a structured rehabilitation program is associated with a statistically significant better outcome (*p* = 0.03) than no rehabilitation.

We observed a higher incidence of TOS among patients working in activities involving intensive computer use, such as video-terminal workers, office workers, teachers (34% of patients); and in activities involving the mobilization of disabled patients, such as social-health workers and/or nurses, subjected to repeated efforts of the upper limbs in an abducted position (12% of patients). Our findings confirm the important role that work activity plays in the development of TOS, in line with what reported in the literature (57, 63). Despite the higher incidence of TOS found in the female sex, we didn't observe sex-related differences in terms of efficacy of rehabilitative treatment, as well as in terms of symptoms improvement.

A situation potentially influencing the outcome of rehabilitative treatment in TOS patients is the presence of concomitant diseases. The rehabilitation program did not result in a better outcome in patients with previous cervical trauma or whiplash, or with supernumerary cervical ribs or C7 anomalies (*p* = 0.4 and *p* = 0.8, respectively). However, significant differences in the rehabilitation outcome were found in patients presenting with musculo-tendon pathologies of the shoulder and TOS vs. patients with TOS but without shoulder pathologies (*p* = 0.04). This difference is likely due to the fact that the rehabilitative pathway performed by the patients is focused only on treating TOS-symptoms.

## Conclusions

The objective diagnosis of TOS is a continuous challenge due to the wide variety of non-specific symptoms and of differential diagnosis. Despite several progresses in the diagnostic process in the last 20 years, significant technical issues and controversies still persist. In this sense, clinical suspicion should be confirmed by objective (instrumental) diagnosis, in order to achieve prompt recognition of the syndrome and a swift start of the treatment for rapid and successful results.

We believe that a structured and standardized rehabilitative process should represent the initial treatment for TOS, leaving surgery only for patients who failed to improve after a conservative management program or with refractory or recurrent symptoms.

Albeit encouraging and in line with the literature, our results require confirmation coming from properly designed studies on a larger patient cohort.

## Data Availability Statement

The raw data supporting the conclusions of this article will be made available by the authors, without undue reservation.

## Ethics Statement

Ethical review and approval was not required for the study on human participants in accordance with the local legislation and institutional requirements. Written informed consent for participation was not required for this study in accordance with the national legislation and the institutional requirements.

## Author Contributions

GC and AV gave substantial contributions to the conception or design of the work. GC, AV, AP, ASc, ASt, FC, PD, AZ, RS, FS, and MP provided the acquisition and analysis or interpretation of data for the work. GC, EB, SM, FR, FG, and PS drafting the work or revising it critically for important intellectual content. GC, AV, AP, ASc, ASt, FC, AZ, FS, MP, RS, EB, PD, SM, FR, FG, and PS provided approval for publication of the content. GC, AV, AP, ASc, ASt, FC, AZ, FS, MP, RS, EB, PD, SM, FR, FG, and PS agree to be accountable for all aspects of the work in ensuring that questions related to the accuracy or integrity of any part of the work are appropriately investigated and resolved. All authors contributed to the article and approved the submitted version.

## Conflict of Interest

The authors declare that the research was conducted in the absence of any commercial or financial relationships that could be construed as a potential conflict of interest.

## Publisher's Note

All claims expressed in this article are solely those of the authors and do not necessarily represent those of their affiliated organizations, or those of the publisher, the editors and the reviewers. Any product that may be evaluated in this article, or claim that may be made by its manufacturer, is not guaranteed or endorsed by the publisher.
