# Corrigendum: Diagnostic and therapeutic management of the thoracic outlet syndrome. Review of the literature and report of an Italian experience

**DOI:** 10.3389/fcvm.2023.1273543

**Published:** 2023-09-13

**Authors:** Giuseppe Camporese, Enrico Bernardi, Andrea Venturin, Alice Pellizzaro, Alessandra Schiavon, Francesca Caneva, Alessandro Strullato, Daniele Toninato, Beatrice Forcato, Andrea Zuin, Francesco Squizzato, Michele Piazza, Roberto Stramare, Chiara Tonello, Pierpaolo Di Micco, Stefano Masiero, Federico Rea, Franco Grego, Paolo Simioni

**Affiliations:** ^1^ Angiology Unit, Department of Cardiac, Thoracic and Vascular Sciences and Public Health, Padua University Hospital, Padua, Italy; ^2^ Department of Emergency and Accident Medicine, Hospital of Treviso, Treviso, Italy; ^3^ Physical Medicine and Rehabilitation Unit, Department of Neurosciences, Padua University Hospital, Padua, Italy; ^4^ Thoracic Surgery, Department of Cardiac, Thoracic and Vascular Sciences and Public Health, Padua University Hospital, Padua, Italy; ^5^ Vascular Surgery, Department of Cardiac, Thoracic and Vascular Sciences and Public Health, Padua University Hospital, Padua, Italy; ^6^ Department of Medicine DIMED, Institute of Radiology, Padua University Hospital, Padua, Italy; ^7^ Unit of Advanced Clinical and Translational Imaging, Department of Medicine, University Hospital of Padua, Padua, Italy; ^8^ Department of Internal Medicine and Emergency Room, Naples Buon Consiglio Fatebenefratelli Hospital, Naples, Italy; ^9^ Department of Internal Medicine, General Medicine Unit, Thrombotic and Haemorrhagic Disorders Unit, University Hospital of Padua, Padua, Italy

**Keywords:** diagnosis, treatment, thoracic outlet syndrome, surgery, rehabilitation

A Corrigendum on Diagnostic and therapeutic management of the thoracic outlet syndrome. Review of the literature and report of an Italian experience By Camporese G, Bernardi E, Venturin A, Pellizzaro A, Schiavon A, Caneva F, Strullato A, Toninato D, Forcato B, Zuin A, Squizzato F, Piazza M, Stramare R, Tonello C, Di Micco P, Masiero S, Rea F, Grego F, Simioni P (2022). Front. Cardiovasc. Med. 9:802183. doi: 10.3389/fcvm.2022.802183

In the published article, there was a mistake in the caption of Figure 1. The correct caption is as follows:

**Figure 1.** Anatomical spaces involved in TOS. Originally published in “The human spring approach to thoracic outlet syndrome”. Author: Dr. James Stoxen DC FSSEMM (hon); illustrated and copyrighted by Body Scientific International, LLC – www.BodyScientific.com. Permission of the Author with agreement signed in February 2022.

The authors apologize for this error and state that this does not change the scientific conclusions of the article in any way. The original article has been updated.

