# Supplementary material for: Diagnostic and Therapeutic Management of the Thoracic Outlet Syndrome. Review of the Literature and Report of an Italian Experience
Source: Front Cardiovasc Med. 2022 Mar 22;9:802183. doi: 10.3389/fcvm.2022.802183 (PMC8983020; doi:10.3389/fcvm.2022.802183)

Supplementary Material

Contents

1. **Ultrasound methodology……………………………………………………….………...2**
2. **Contrast-enhanced Computed Tomography Angiography methodology………2**
3. **Contrast-enhanced Magnetic Resonance Angiography methodology…………..3**
4. **Conservative rehabilitative treatment…………………………………………….3**
5. **Trans-axillary surgical approach…………………………………………………4**
6. **Supraclavicular surgical approach………………………………………………4**
7. **Table 1S. List of signs and symptoms according to the type of TOS……….…..5**
8. **Table 2S. List of provocative maneuvers for clinical diagnosis of TOS………..6-7**
9. **Table S3. Exercises for the muscles of the shoulder……………….…………….…7**
10. **Table S4. NRS-score distribution at T0, T1, and T2…………………..……………..8**
11. **Table S5. Outcomes in patients with shoulder disease…………….……………..9**
12. **Table S6. Outcomes in patients with rehabilitation program versus those undergoing surgery by NRS score…………………………………………………………10**
13. **Figure 1S. CTA reconstruction of a right subclavian artery in a patients with aTOS and acute limb ischemia. Note the presence of embologenic thrombus at the level of the arterial compression…………………………………….……..………..11**
14. **Figure 2S. MRA showing chronic occlusion of the right subclavian artery in a patient with aTOS…………………………………………………………...…………12**
15. **Figure 3S. DSA of a patient admitted with acute upper extremity ischemia related to aTOS…………………………………………………………………..……………...13**
16. **Figure 4S. Stretching of the subclavian, pectoral and scalene muscles, mobilizations and breathing exercises………………………...………………….....………..14**
17. **Figure 5S. Xray after right first rib resection (red arrow)…..……………………..15**

METHODOLOGY

1. Ultrasound methodology

First, with the patient supine, deep-vein thrombosis of the subclavian, axillary and brachial veins is ruled-out by compression ultrasonography. If deep-vein thrombosis is excluded, the patient is allowed to sit with the back and neck straight, and with the shoulders resting in a neutral position. The probe is then placed longitudinally under the clavicle and the pulse repetition frequency is lowered to a slow-flow velocity program for vein flow detection. Once normal venous flow is detected, provocative maneuvers are started, with the arm being passively abducted at 90° and 180°. A nurse standing behind the bed supports the patient's arm during the maneuvers, so as to avoid muscle contraction, potentially altering blood-flow dynamics. The finding of a "paradoxical obstruction" of the deep venous outflow, characterized by a complete flow-stop of and by a dilatation of the subclavian vein proximal to the compression site is diagnostic for significant vein compression (vTOS). Arterial flow is investigated at the axillary artery, because the subclavian artery runs below the clavicle and, especially during the provocative maneuvers, it is difficult to insonate that vessel properly. As well as for the venous investigation, the axillary artery is explored with the arm abducted at 90° and 180°. A significant compression of the subclavian artery translates in a damped Doppler waveform (monophasic or biphasic), or in a complete Doppler waveform loss recorded in the axillary artery, according to the grade of compression.

2. Contrast-enhanced Computed Tomography Angiography methodology

The contrast-enhanced CT-angio study is carried out using a total dose of 1.5 mL/kg of contrast medium, injected at a speed of 4 mL/s with an automatic injector, in the opposite arm to that showing the main symptoms. Generally, the contrast medium is injected as two half-dose boluses, each of those followed by a matching bolus of physiological solution. This two-step method of administering the contrast medium improves the imaging of the venous district, especially during acquisition of data with hyper-abducted arms. The examination involves a multiphase technique, with bolus tracking, the region of interest being located at the aortic arch, and the threshold set at 70 UH for data acquisition during the arterial phase. Data are acquired in the venous phase at 60 s and 75-85 s (after hyperabduction of the arms), optimized by two-phase injection of contrast medium, thus reducing the time between contrast medium injection and the image acquisition. Coronal, sagittal and 3-D multiplanar reconstructions of the thoracic outlet are obtained with volume rendering techniques to allow simultaneous analysis of both bony and vascular structures. CT-angio, including time required for preparing and positioning the patient, takes about 10 minutes to complete; processing of multiplanar and 3-D reconstructions requires about another 10 minutes. In particular, coronal and sagittal reconstructions facilitate identification of the locus and the severity of stenosis, with visualization of the transverse diameter of vessels and comparison with portions proximal and distal to it, before and after elevation of the upper arm.

3. Contrast-enhanced Magnetic Resonance Angiography methodology

The MRA imaging protocol for TOS is designed to evaluate the vascular tree and is performed with gadolinium-based intravenous contrast material. The examination can be performed on a 1.5-T or 3-T scanner; thus, the image acquisition parameters should be adjusted according to the type of equipment. Surface and phased body array receiving coils are used. The MRA protocol takes a total of about 40 minutes to be completed and includes sequences with breath holding; axial, coronal and sagittal T1 gradient-echo flash sequences; T2 turbo spin-echo and axial turbo inversion recovery magnitude (TIRM) sequences; time of flight (TOF) angiographic sequences; and sagittal true fast imaging with steady-state-free precession (TRUFI) sequences. The axial T1, TOF and sagittal TRUFI sequences are repeated in hyperabduction For contrast-enhanced MRA, 20 mL of a gadolinium-based contrast agent can be used with a 20-mL saline flush at a rate of 2 mL/s during abduction positioning. Moreover, 15 mL of contrast agent should be administered for the same contrast-enhanced sequences in the rest position. The timing of the MRA acquisition can be determined by automatic triggering at contrast agent arrival. For each arm positioning, 2 sets of coronal oblique 3D sets should be acquired. The gadolinium-based contrast agents shorten T1 relaxation time of the blood and signal of the vascular contrast is independent of flow dynamics. Fast and ultra-fast imaging and a large field of view with high spatial resolution in a single hold of breath should be performed. Typically, the less symptomatic arm is used for injection and contrast-enhanced imaging (3D-MRA and volume interpolated gradient echo) is performed in abduction first, with identical sequences repeated in adduction. Before and after hyperabduction of the arms, at the level of the examined anatomical spaces, the following measures should be taken: pre-scalene (diameter of the subclavian vein), interscalene (diameter of the subclavian artery and maximum thickness of the anterior scalene muscle), costoclavicular (minimum costoclavicular distance, distance between posterior border of the subclavian muscle and the superior margin of the first rib, diameters of the subclavian artery and vein, maximum thickness of the subclavian muscle), subchoracoid (diameters of the axillary artery and vein, maximum thickness of the retropectoralis minor muscle) and calipers of the axillary artery and vein.

4. Conservative rehabilitative treatment

Firstly, it is necessary to work on the stabilizing muscles of the scapula with shoulder range of motion from 0°- 30° of flexion and 0°-40° of abduction, then increase to 45°- 90° of flexion and finally overhead exercises. Secondly, exercises against resistance of the muscles that open the thoracic strait (trapezius, rhomboid, grand dorsal and elevator of the scapula) can be performed with elastic bands or dumbbells, with low weight and high number of repetitions. During treatment, focus should be placed on stretching the scalene, pectoral, and subclavian muscles and strengthening the muscles of the cervical spine (i.e., cervical erectors, greater and lesser rhomboid, and lower trapezius)

5. Trans-axillary surgical approach

In this approach the patient, under general anesthesia, is placed in the lateral position with the involved arm, pre-wrapped to allow a gentle traction during the procedure, abducted at 90°. A transverse incision is made below the hairline between the pectoralis major and the latissimus dorsi muscles and dissected to the external intercostal fascia. Careful dissection of the neurovascular bundle is performed and its relation to the first rib and both scalene muscles is clearly identified to avoid injury to these structures. The insertion of the anterior scalenus muscle on the first rib is dissected and the muscle is divided; the first rib is dissected subperiosteally and carefully separated, if possible, from the underlying pleura, the costoclavicular ligament is cut and the first rib can be divided. Then, the anterior portion is gently pulled away to decompress the anterior venous compartment, while the posterior segment is dissected from the subclavian artery and the brachial plexus and is divided from the transverse vertebral process. It is preferable to remove the entire first rib, including its head and neck, to avoid future irritation of the plexus, because a residual portion may cause recurrence of symptoms. The eighth cervical and first thoracic nerve roots can be clearly visualized at this point and, if a cervical rib is present, it is removed at this time and the seventh cervical nerve root can be observed. Then the patient is encouraged to use the arm normally and can be discharged from the hospital between 2 and 3 days after the surgical procedure.

6. Supraclavicular surgical approach

This approach releases soft-tissue compressive structures in the interscalene portion of the brachial plexus. The lower trunk and root of C8 and T1 can be completely identified and protected while the most posterior aspect of the first rib is resected under direct vision.
After general anesthesia, the patient is positioned supine with the head and the neck extended and elevated 30°, then a 6–8 cm supraclavicular incision is made. After cutting the platysma, the supraclavicular nerves are identified and protected, the supraclavicular fat pad is elevated from the lateral side of internal jugular vein and the phrenic nerve is seen on the anterior surface of the anterior scalene muscle, usually running from lateral to medial, that is divided from the first rib: the subclavian artery lays immediately behind.
Now, after the division of the middle scalene muscle from the first rib, the upper, middle and lower trunks of the brachial plexus are easily visualized, gently mobilized and neurolysis is performed. If a cervical or an anomalous rib is present in this anatomical plane, it may be removed at this point.

TABLES

Table 1S. List of signs and symptoms according to the type of TOS

TOS	Anamnesis	Signs and symptoms
nTOS<br><br>cervical plexus compression	Chronic worsening onset	pain<br><br>paresthesia
	Repetitive movements	weakness and atrophy of thenar, hypothenar and interosseous muscles eminence, loss of dexterity
	Athletes	occipital headache
	Cervical spine trauma	altered tone of trapezius, scalene, pectoralis major/minor, elevator of the scapula, sternocleidomastoid, serratus anterior muscles<br><br>Superior plexus (C5-C7): pain in the neck, shoulder, chest and supraclavicular region, arm weakness, paresthesia of the I, II and III finger;<br><br>Inferior plexus (C7-T1): pain in the medial region of the arm, forearm and hand, paresthesia IV- V finger
vTOS<br><br>subclavian vein compression	Acute onset<br><br>Upper limb vein thrombosis<br><br>Exacerbated by lifting weights above the head.	upper limb edema<br><br>cyanosis<br><br>feeling of heaviness in the arm<br><br>pain<br><br>temperature alteration
aTOS<br><br>subclavian artery compression	Cervical rib<br><br>Acquired in professional weightlifters	Chronic:<br><br>pain<br><br>reduction or disappearance of radial pulse following arm movements<br><br>paleness<br><br>pulsations, blowing in the supraclavicular area (aneurysm)<br><br>difference of blood pressure between the limbs<br><br>weakness and fatigue

		temperature alteration<br><br>Acute: ischemia of the hand in case of distal embolization

Table 2S. List of provocative maneuvers for clinical diagnosis of TOS

Test	Procedure	Positive Test
**Adson Test**	The examiner monitors the radial pulse at wrist while the patient's arm is held in extension, the shoulder in external rotation and 30° abduction. Patient takes a deep breath and turns head toward the test arm while extending the neck.	Decrease/disappearance of radial pulse.
**Modified Adson Test**	The examiner locates the radial pulse, the shoulder is abducted at 90° with extended elbow. Patient takes a deep breath and turn head away from the side tested	Decrease/disappearance of radial pulse.
**Upper Limb Neural Tension Test (ULTT)**<br><br>**or modified test of Elvey**	Shoulder girdle depression, shoulder abduction and external rotation, elbow flexion, wrist and Finger extension	Onset of paresthesia
**Elevated Arm Stress Test (EAST)**<br><br>**Roos Test**	Patient abducts shoulders to 90 °, externally rotates the shoulders, and flexes the elbows to 90°. Then he opens and closes the hand slowly for three minutes.	Inability to complete the test or experiences heaviness, numbness, tingling or pain
**Halstead Maneuver**	The examiner palpates the radial pulse and applies downward traction on the test extremity while the patient's neck is hyperextended and rotated to the opposite side.	Decrease/disappearance of radial pulse.

**Eden Test**	The patient is sitting with the back straight and the shoulders pushed backward and downward. The examiner depresses the shoulder while monitoring the radial pulse at the wrist.	Decrease/disappearance of radial pulse.
**Wright Test**	The examiner brings the arm in abduction and external rotation to 90° without tilting the head. The elbow is flexed no more than 45°. The arm is then held for 1 min.	Decrease/disappearance of radial pulse. Onset of pare-sthesia

Table S3. Exercises for the muscles of the shoulder

EXERCISES	MUSCLES
Scapular Retraction	Rhomboids, Trapezius
Prone shoulder extension, abduction, horizontal abduction	Rhomboids, Trapezius, Supraspinatus, Infraspinatus, Deltoid, Latissimus dorsi, Large round
Scapular depression	Trapezius (lower bundles), Latissimus dorsi, Rhomboids
Frontal raises	Deltoid
Standing external rotation	Trapezius (lower bundles), Infraspina-tus, Small round, Subscapularis
Lateral raises	Deltoid, Supraspinatus, Trapezius
Straight arm extension	Latissimus dorsi, Small round, Triceps brachii
Serratus push	Serratus Anterior

Banded high rows	Latissimus dorsi, Trapezius, Rhomboids, Small and large round
Chin tuck	Trapezius

Table S4. NRS-score distribution at T0, T1, and T2.

		Treatment		
	NRS score	NO (39)	Yes (285)	p
t-0	4	2 (5.1)	14 (4.9)	0,965
	5	7 (17.9)	52 (18.2)	
	6	7 (17.9)	62 (21.8)	
	7	8 (20.5)	44 (15.4)	
	8	9 (23.1)	74 (26)	
	9	6 (15.4)	39 (13.7)	
t-1	1	0 (0)	1 (0.4)	0,192
	2	0 (0)	1 (0.4)	
	3	0 (0)	12 (4.2)	
	4	4 (10.3)	44 (15.4)	
	5	8 (20.5)	66 (23.2)	
	6	12 (30.8)	92 (32.3)	
	7	7 (17.9)	46 (16.1)	
	8	8 (20.5)	19 (6.7)	
	9	0 (0)	4 (1.4)	
t-2	1	0 (0)	1 (0.4)	0,003
	2	0 (0)	2 (0.7)	
	3	2 (5.1)	15 (5.3)	
	4	9 (23.1)	71 (24.9)	
	5	7 (17.9)	86 (30.2)	
	6	6 (15.4)	68 (23.9)	
	7	7 (17.9)	30 (10.5)	
	8	7 (17.9)	12 (4.2)	
	9	1 (2.6)	0 (0)	

Table S5. Outcomes in patients with shoulder disease

Presence of shoulder disease					
YES (39)			NO (246)		
Improved	33	84.6%	Improved	174	70.8%
Stationary	6	15.4%	Stationary	51	20.7%
Worsened	0	0%	Worsened	21	8.5%

Table S6. Outcomes in patients with rehabilitation program versus those undergoing surgery by NRS score

	Specific rehabilitation protocol, n = 256	TOS surgery n = 29	P
Improved	186 (75%)	20 (69%)	0.49
Stationary	64 (25%)	9 (31%)	0.47
Worsened	0	0	--

FIGURES LEGENDS

Figure 1S. CTA reconstruction of a right subclavian artery in a patients with aTOS and acute limb ischemia. Note the presence of embologenic thrombus at the level of the arterial compression

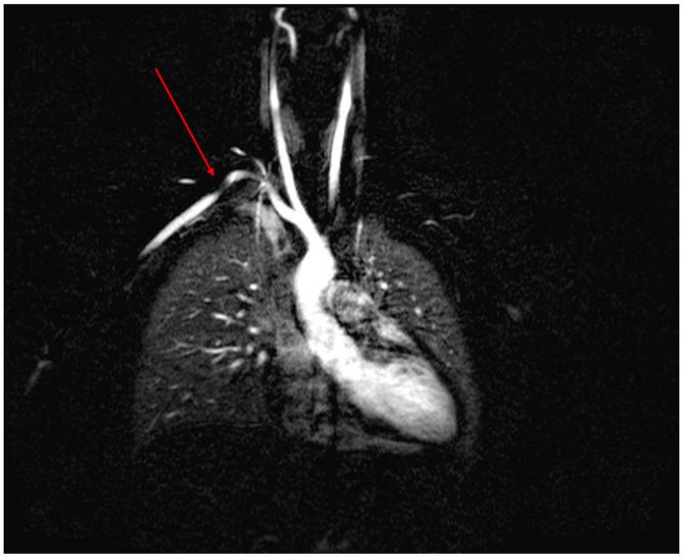

Figure 2S. MRA showing chronic occlusion of the right subclavian artery in a patient with aTOS

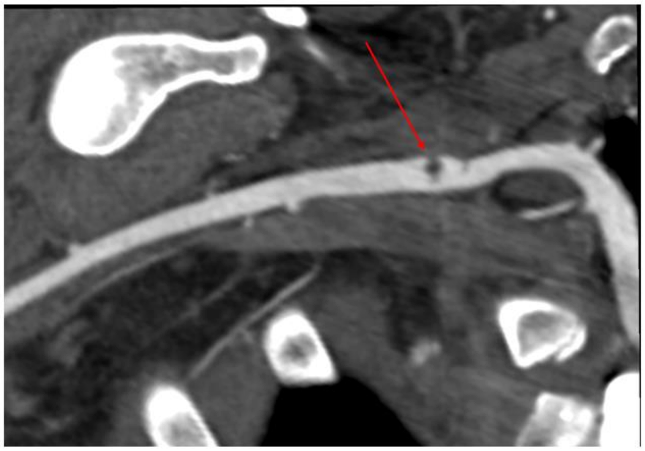

Figure 3S. DSA of a patient admitted with acute upper extremity ischemia related to aTOS

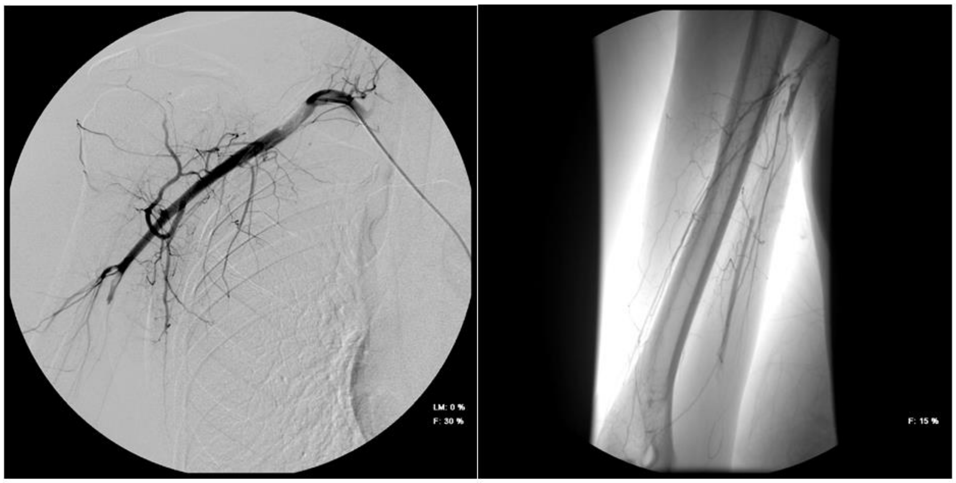

Figure 4S. Stretching of the subclavian, pectoral and scalene muscles, mobilizations and breathing exercises

Stretching of the Scalenus muscle 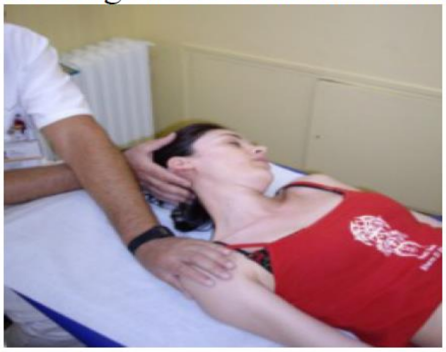	Stretching of Subclavius muscle 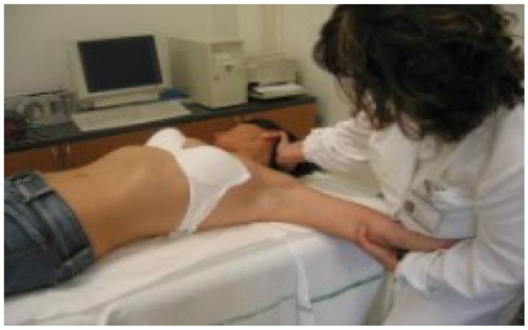
Stretching of pectoralis minor muscle 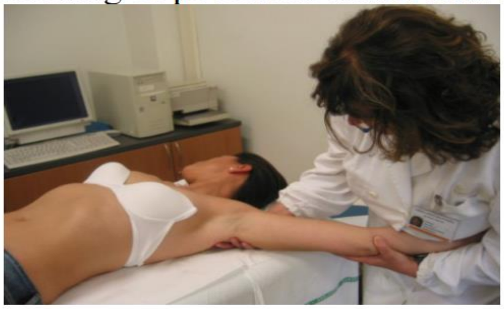	Stretching of subclavius and scalene muscles 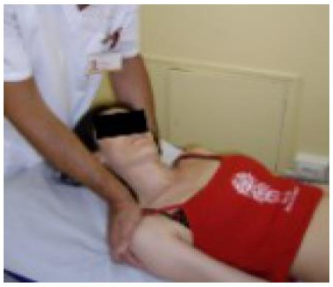
Stretching of Trapezius and posterior scalene muscles 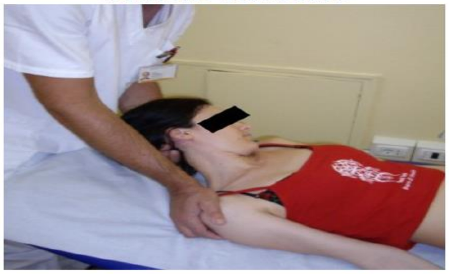	Cervical mobilization 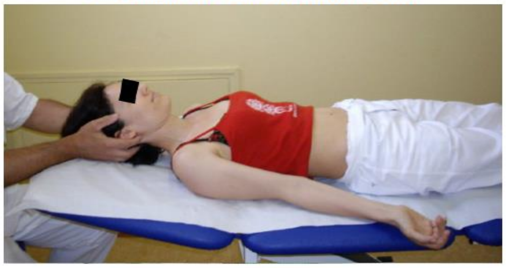
First Rib mobilization (Maitland) 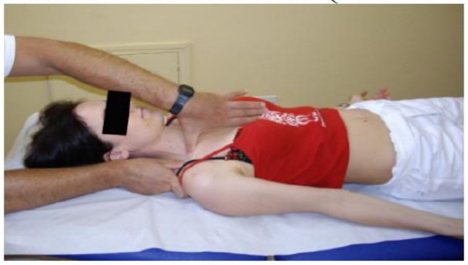	Respiratory exercise 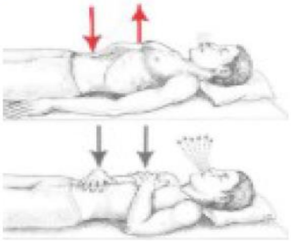

Figure 5S. Xray after right first rib resection (red arrow)

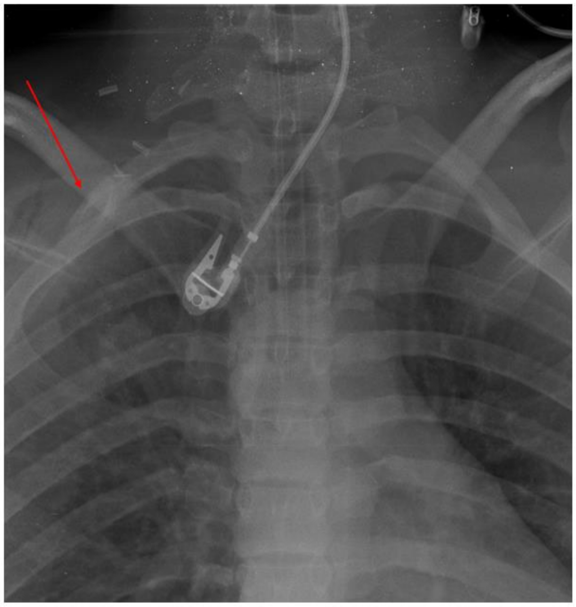

Supplement: Supplementary file 1 [file Data_Sheet_1.pdf]
